# Immunogenicity and protective efficacy of a live, oral cholera vaccine formulation stored outside-the-cold-chain for 140 days

**DOI:** 10.1186/s12865-020-00360-1

**Published:** 2020-05-25

**Authors:** Tew Hui Xian, Kurunathan Sinniah, Chan Yean Yean, Venkateskumar Krishnamoorthy, Mohd Baidi Bahari, Manickam Ravichandran, Guruswamy Prabhakaran

**Affiliations:** 1 Department of Biotechnology, Faculty of Applied Sciences, AIMST University, 08100 Semeling, Kedah Malaysia; 2 Department of Medical Microbiology and Parasitology, School of Medical Sciences, Universiti Sains Malaysia, 16150 Kubang Kerian, Kelantan Malaysia; 3 Faculty of Pharmacy, AIMST University, 08100 Semeling, Kedah Malaysia; 4 Centre of Excellence for Omics-Driven Computational Biodiscovery, Faculty of Applied Sciences, AIMST University, 08100 Semeling, Kedah Malaysia

**Keywords:** Live attenuated, *Vibrio cholerae* O139, Thermostable vaccine, RITARD, Rabbit ileal loop, Reactogenicity

## Abstract

**Background:**

Cholera, an acute watery diarrhoeal disease caused by *Vibrio cholerae* serogroup O1 and O139 across the continents. Replacing the existing WHO licensed killed multiple-dose oral cholera vaccines that demand ‘cold chain supply’ at 2–8 °C with a live, single-dose and cold chain-free vaccine would relieve the significant bottlenecks and cost determinants in cholera vaccination campaigns. In this direction, a prototype cold chain-free live attenuated cholera vaccine formulation (LACV) was developed against the toxigenic wild-type (WT) *V. cholerae* O139 serogroup. LACV was found stable and retained its viability (5 × 10^6^ CFU/mL), purity and potency at room temperature (25 °C ± 2 °C, and 60% ± 5% relative humidity) for 140 days in contrast to all the existing WHO licensed cold-chain supply (2–8 °C) dependent killed oral cholera vaccines.

**Results:**

The LACV was evaluated for its colonization potential, reactogenicity, immunogenicity and protective efficacy in animal models after its storage at room temperature for 140 days. In suckling mice colonization assay, the LACV recorded the highest recovery of (7.2 × 10^7^ CFU/mL) compared to those of unformulated VCUSM14P (5.6 × 10^7^ CFU/mL) and the WT O139 strain (3.5 × 10^7^ CFU/mL). The LACV showed no reactogenicity even at an inoculation dose of 10^4^–10^6^ CFU/mL in a rabbit ileal loop model. The rabbits vaccinated with the LACV or unformulated VCUSM14P survived a challenge with WT O139 and showed no signs of diarrhoea or death in the reversible intestinal tie adult rabbit diarrhoea (RITARD) model. Vaccinated rabbits recorded a 275-fold increase in anti-CT IgG and a 15-fold increase in anti-CT IgA antibodies compared to those of rabbits vaccinated with unformulated VCUSM14P. Vibriocidal antibodies were increased by 31-fold with the LACV and 14-fold with unformulated VCUSM14P.

**Conclusion:**

The vaccine formulation mimics a natural infection, is non-reactogenic and highly immunogenic in vivo and protects animals from lethal wild-type *V. cholerae* O139 challenge. The single dose LACV formulation was found to be stable at room temperature (25 ± 2 °C) for 140 days and it would result in significant cost savings during mass cholera vaccination campaigns.

## Background

Cholera, an acute watery diarrhoeal disease caused by the waterborne bacterium *Vibrio cholerae* is endemic and epidemic in 69 low-and middle-income countries (LMICs) [1–3]. It is predominantly caused by *V. cholerae* serogroups O1 El Tor, and in Asian countries, mostly by O139 [4–6] and the evolution of new toxigenic strains remains an important global health challenge [7–9]. The clinical manifestations of cholera caused by *V. cholerae* O1 versus O139 are indistinguishable. Vaccination is a preventive measure and killed oral cholera vaccines for O1 and O139 and a live vaccine for O1 are available. Markedly, the vaccine for O1 does not cross-protect against cholera caused by O139 and vice versa [10–14]. Killed vaccines confer short-term protection and require a booster dose as opposed to a single-dose live attenuated vaccine that mimics natural infection and eliminates repetitive dosing [15–18]. Although all the existing WHO licensed cholera vaccines are safe, they demand a cold chain supply (2–8 °C) distribution system from manufacturing to the immunization site to ensure their safety and potency and cold chain logistics are difficult to execute in LMICs [19, 20]. Hence, these mandatory requirements resulted in a high cost of vaccination which poses a great challenge [21, 22]. A cold chain free version of any cholera vaccine would relieve the bottlenecks and cost determinants and result in significant cost savings during mass vaccination campaigns [23–25]. Therefore, it is inevitable to develop a single dose and cold chain free live cholera vaccine.

Live cholera vaccine candidates have been developed by attenuation of virulence in the pathogenic strains by genetic engineering. However, in the development of a live attenuated cholera vaccine candidate, the degree of attenuation has been hampered by its unacceptable clinical side effects or reactogenicity (adverse reactions) in volunteers, such as a headache, vomiting, diarrhoea, including noncholeric diarrhoea and abdominal cramps, which are a cause of concern when compared to the vaccines formulated with heat-killed cells [26, 27]. Hence, the vaccine candidate must be genetically stable, immunogenic and unable to revert to the pathogenic phenotype. Towards this, several live attenuated vaccines against O1 and O139 are in various stages of development and evaluation with the vaccine candidates CVD-103 HgR [28, 29], VA1.3/VA1.4 [30], Peru-15 [31], IEM 101/108/109 [32], Cuban 638 [33, 34], Texas Star [35] and Wzm [36], CVD112 [37], Bengal-15 [38], TLP01 [39], VRI-16 [40] and L911/L912 [41]. However, similar to killed cholera vaccines, live vaccine formulations are also heat-sensitive and cold chain supply dependent. Therefore, a cold chain-free, live, attenuated cholera vaccine that can be stored at room temperature must be developed to increase its outreach to global immunization programmes.

Notably, there is no live vaccine exclusively available to protect against cholera caused by *V. cholerae* O139 and to date, no cold chain-free live attenuated oral cholera vaccine against O1 and O139 has been commercialized. In this direction, live attenuated aminolevulinic acid (ALA) auxotroph VCUSM1 and VCUSM2 strains protective against *V. cholerae* O139 were constructed by frameshift mutation of a housekeeping gene, *hem*A that encodes for glutamyl-tRNA reductase, an important enzyme in the C5 pathway for delta-aminolevulinic acid (ALA) biosynthesis, which renders this strain dependent on exogenous ALA for survival and these vaccine candidates were patented [42, 43]. The VCUSM2 was found to be immunogenic but showed mild reactogenic effects in animal models due to the presence of two copies of cholera toxin genetic element. Hence, this strain was further improved by mutating the *ctx*A gene via substitution of 7th amino acid, arginine to lysine, R7K, and 112th amino acid, glutamate to glutamine, E112Q. And, two copies of *ace* and *zot* genes in *ctx* operon were also deleted to reduce the reactogenicity and resultant strain VCUSM14 (an aminolevulinic acid (ALA) auxotrophic and non-reactogenic) was characterised and evaluated in animal models [44]. Further, the *hem*A gene was reintroduced into VCUSM14 to enhance its colonization potential, and the strain was designated as VCUSM14P. The VCUSM14P is a non-toxigenic strain and an aminolevulinic acid (ALA) prototroph with enhanced colonization and immunogenic properties against infection by the *V. cholerae* O139 serogroup.

The development of cold chain free, live liquid vaccine formulation was based on the understanding of the survival mechanisms of both toxigenic and nontoxigenic *V. cholerae* strains that inhabit nutrient-poor, temperature and oxygen fluctuating levels in aquatic ecosystems year-round. These adaptive traits of *V. cholerae* were leveraged at an in-vitro condition that best mimic the environmental stress conditions for the extended storage period of live attenuated cholera vaccine strain VCUSM14P at room temperature (25 °C ± 2 °C). Eventually, a prototype cold chain-free, live-attenuated oral cholera vaccine (LACV) formulation with VCUSM14P was developed and a ‘Patent Filed’. The LACV is a liquid formulation consisting of 5 × 10^6^ CFU/mL of the VCUSM14P strain. Accelerated storage stability studies were performed for six batches of LACV in an ICH-compliant stability chamber (Binder-KBF 115, Germany) at 25 °C ± 2 °C and 60% ± 5% relative humidity (RH). At fixed intervals, the viability of the VCUSM14P strain in the LACV was enumerated and its genetic purity was ascertained by polymerase chain reaction. The statistical analysis was performed by using the Wilcoxon Rank Sum Test and no significant differences observed between the batches. The LACV was found stable at 25 °C ± 2 °C and 60% ± 5% RH by retaining its viability, purity and potency for 140 days. After 140 days, the cell viability was decreased by one to two orders of magnitude and hence the LACV was not tested further for its safety and efficacy. The repeated dose toxicity of the LACV was evaluated in Sprague Dawley (SD) rats to ascertain its safety for clinical use [45]. The present study focused on the evaluation of colonization potential, reactogenicity, immunogenicity and protective efficacy of the LACV after its storage at room temperature for 140 days in animal models for the development of a single-dose, cold chain-free, live oral cholera vaccine against *V. cholerae* O139.

## Results

### Infant mouse colonization assay

**Fig. 1:**
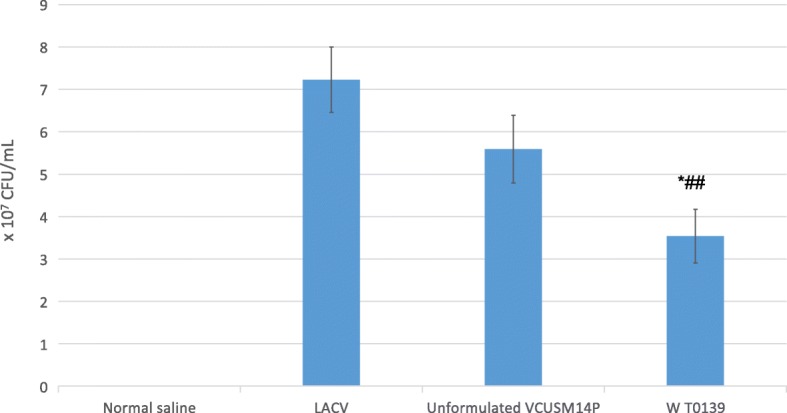
Suckling mouse intestinal colonization potential of the normal saline, LACV, unformulated VCUSM14P and the WT O139 strain. All the values are Mean ± SD (*n* = 10). *P < 0.05 compared with LACV. ##*P* < 0.01 compare with Unformulated VCUSM14P. One-way ANOVA followed by Tukey *post-hoc* test

Colonization of vibrios on the mucosal layer of the small intestine is critical for elicitation of a protective immune response. We compared the colonization potential of the LACV with that of unformulated VCUSM14P and the WT O139 strain. The LACV recorded the highest average recovery, with more cells (7.2 × 10^7^ CFU/mL) recovered from the intestine than from unformulated VCUSM14P (5.6 × 10^7^ CFU/mL) and the WT O139 strain (3.5 × 10^7^ CFU/mL) (Fig. 1).

### Rabbit ileal loop reactogenicity assay

**Fig. 2:**
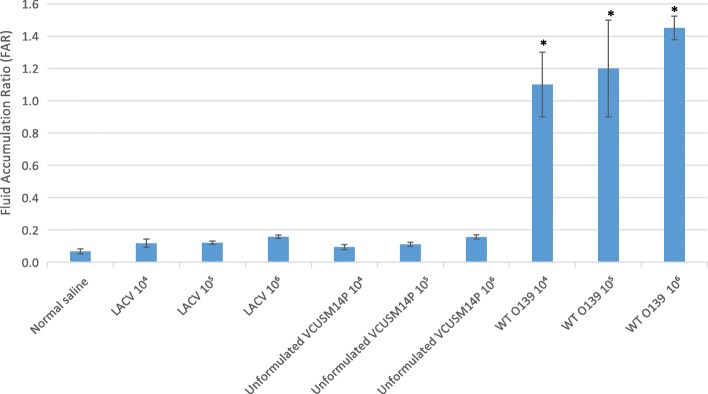
Fluid accumulation ratio (FAR) in ligated ileal loops injected with the normal saline, LACV, unformulated VCUSM14P and the WT O139 strain in unvaccinated rabbits. All the values are Mean ± SD (*n* = 3). *P < 0.05 compared with Normal saline. One-way ANOVA followed by Tukey post-hoc test

**Fig. 3:**
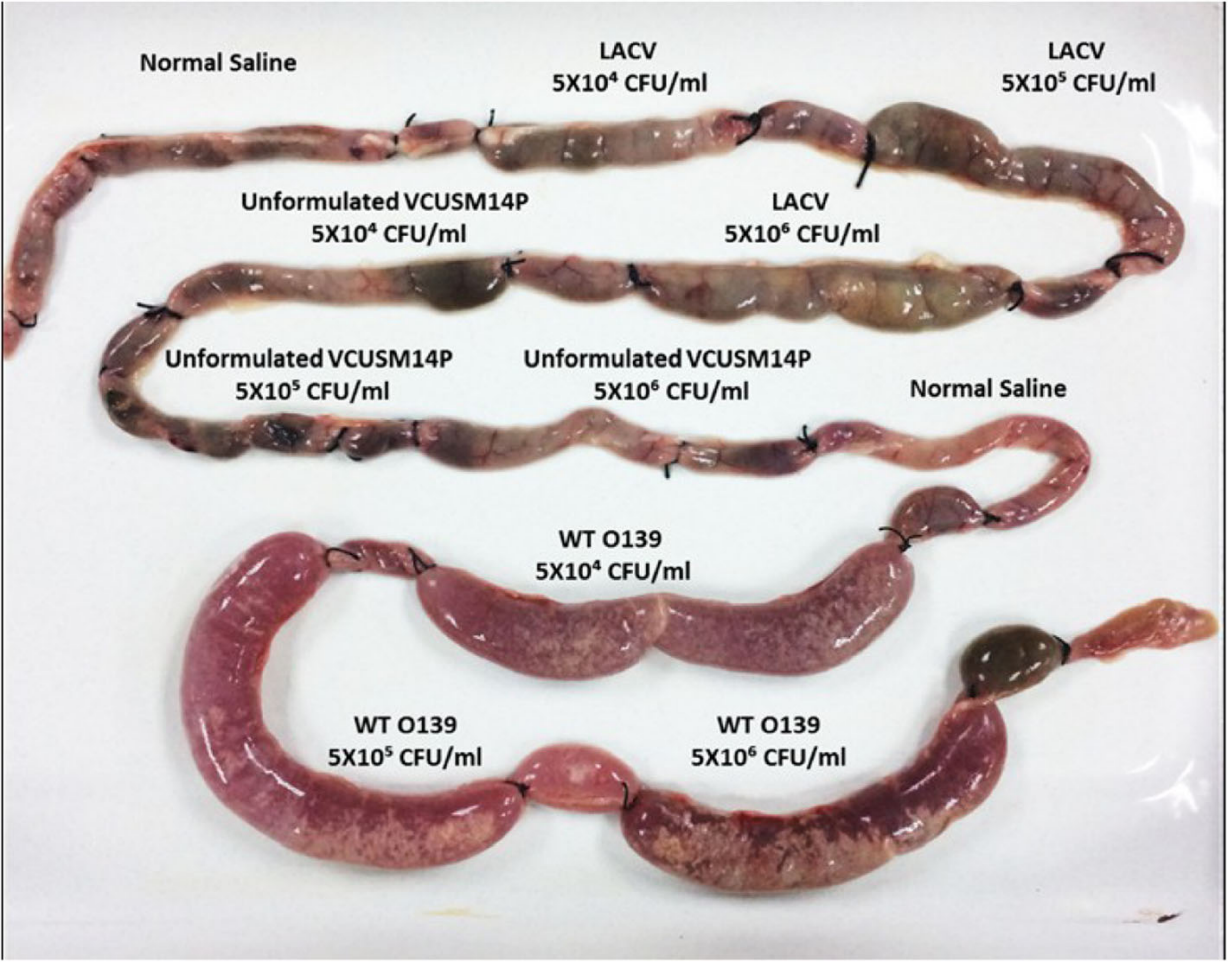
Ileal loops injected with the LACV, unformulated VCUSM14P and WT O139 in an unvaccinated rabbit showing the presence or absence of fluid accumulation and haemorrhage

In the ligated ileal loop assay, loops injected with 1 mL of the LACV or unformulated VCUSM14P were recorded with less than 0.2 fluid accumulation ratio (FAR). In contrast, the loops injected with 1 mL of the WT O139 strain exhibited a 4-fold increase in FAR with the symptoms of acute cholera. Besides, the presence of haemorrhage was also observed in the loops, showing necrosis in the intestinal loops (Figs. 2 and 3). Distinctly, no fluid accumulation and haemorrhage were observed in the loops injected with the LACV and unformulated VCUSM14P.

### Protective efficacy in the RITARD (reversible intestinal tie adult rabbit diarrhoea) model

There was 100% mortality of the unvaccinated rabbits within 24 h with obvious fluid accumulation and haemorrhage in the small intestine. The vaccinated rabbits did not show any signs of diarrhoea, mortality or fluid accumulation in the small intestine for up to 5 days of observation (Table 1).

**Table 1:** Evaluation of the protective efficacy of the LACV and unformulated VCUSM14P in the RITARD model

Challenge outcome	Rabbit orally vaccinated with the LACV (***n*** = 3)	Rabbit orally vaccinated with the VCUSM14P strain (***n*** = 3)	Unvaccinated rabbit (***n*** = 3)
**Mild diarrhoea**	0 of 3	0 of 3	0 of 3
**Severe diarrhoea**	0 of 3	0 of 3	3 of 3
**Intestinal h**a**emorrhage**	0 of 3	0 of 3	3 of 3
**Animal death**	0 of 3	0 of 3	3 of 3
**Percent mortality**	0%	0%	100%
**Fluid accumulation in the small intestine**	No	No	Yes

### Bacterial shedding after vaccination

The shedding of VCUSM14P in the rectal swabs of immunized rabbits indicates intestinal colonization and multiplication of the vaccine candidate in the intestine. All vaccinated rabbits shed VCUSM14P for 3 days after the first vaccination. However, no shedding of vibrios was observed after the booster dose administered in any vaccinated rabbits. In rectal swabs, the vibrios were recovered for up to 3 days in the vaccinated rabbits that were challenged with the WT O139 strain.

### Immunological analysis

The immune response of vaccinated rabbits was determined by measuring the anti-CT IgG, anti-CT IgA and vibriocidal antibodies. The enzyme-linked immunosorbent assay (ELISA) results showed a significant increase in anti-CT IgG and anti-CT IgA titres in LACV-vaccinated rabbits after the first dose of vaccination. After the booster dose on week 2, there was a further increase in the titre, and it peaked on week 4 for anti-CT IgG (275-fold) and anti-CT IgA (15-fold). In comparison, only on week 4, there was a 7.9-fold increase in anti-CT IgA, and a 64-fold increase in anti-CT IgG in the rabbits vaccinated with unformulated VCUSM14P.

In the present study, the pre-immune sera of unvaccinated rabbits showed low vibriocidal antibody titres of 10. On the second week of vaccination with the LACV or unformulated VCUSM14P, there was a 4-fold increase in vibriocidal antibody titre when compared to that of the basal titre of 10 in pre-immune serum. After the fourth week, there was a 31-fold increase in vibriocidal antibodies in the rabbits vaccinated with the LACV. In comparison, there was only a 14-fold increase in vibriocidal antibodies in the rabbits vaccinated with unformulated VCUSM14P (Table 2).

**Table 2:** Geometric mean titre (GMT) of anti-CT IgG, anti-CT IgA and vibriocidal antibodies elicited in rabbits vaccinated with either the LACV or unformulated VCUSM14P

Immune response (***n*** = 3)		GMT (range) on week				
		Pre	First	Second	Third	Fourth
**Anti-CT IgG**	LACV	10.00 (10)	17.12 (16–22)	697.17 (531–829)	1585.87 (1270–2307)	2754.71 (2351–3191)
	Unformulated VCUSM14P	10.00 (10)	15.44 (12–18)	162.66 (138–182)	382.00 (375–387)	646.18 (610–712)
**Anti-CT IgA**	LACV	10.00 (10)	72.67 (65–78)	94.87 (88–100)	129.00 (114–151)	150.95 (143–161)
	Unformulated VCUSM14P	10.00 (10)	67.39 (61–73)	77.77 (63–86)	86.78 (85–90)	79.82 (64–90)
**Vibriocidal antibody**	LACV	10.00 (10)	40.00 (40)	153.03 (140–160)	156.59 (150–160)	316.42 (300–330)
	Unformulated VCUSM14P	10.00 (10)	40.00 (40)	139.04 (120–160)	149.30 (130–160)	145.37 (120–160)

## Discussion

A single-dose cold chain-free cholera vaccine would cost less due to its storage and distribution at room temperature. An ideal live attenuated oral cholera vaccine should colonize effectively, be non-reactogenic and immunogenic and induce a protective immune response against a challenge. Therefore, this study focused on the evaluation of a LACV for these key attributes. Intestinal colonization is a pre-requisite for the establishment of infection and subsequent elicitation of the immune response [46–48]. It was proposed that an inoculum containing *V. cholerae* 10^3^–10^8^ cells is required for an effective infection of a human host [49]. In agreement with this hypothesis, in the present study, we found an increased colonization potential of the LACV at a dose of 2.5 × 10^5^ CFU/50 μL in infant mice. Similar to our results, a dose of 10^5^ CFU of *V. cholerae* (El Tor strain C6706) was effective in colonization and biofilm development in infant mice, as reported in [50]. In contrast, the VCUSM2, VCUSM14 and VCUSM21P strains were recovered with 1.8 × 10^5^ CFU, 2.0 × 10^4^ CFU and 1.55 × 10^6^ CFU respectively, from mouse intestines [44, 51]. In comparison to the recovery of these VCUSM strains, a two-log higher recovery of VCUSM14P was recorded in the present study. The higher colonization potential of the LACV could be due to the mutation in *ctx*A, deletion of the *ace* and *zot* genes and the presence of the *hem*A gene in the vaccine (VCUSM14P) strain.

The safety of a live attenuated cholera vaccine is mainly focused on its reactogenicity. Reactogenicity (adverse reactions) of a live attenuated cholera vaccine in volunteers is a cause of concern [52] and dependent on *V. cholerae* flagellins [27]. Reactogenicity of a cholera vaccine has been assessed based on the fluid accumulation ratio (FAR) in rabbit ligated ileal loops. A FAR greater than 1.0 indicates a strong toxigenicity of cholera toxin and less than 0.2 indicates no reactogenicity [53, 54]. In unvaccinated rabbits, the loops injected with the LACV and unformulated VCUSM14P were recorded with less than a 0.09–0.16 FAR. In contrast, the loops injected with WT O139 exhibited a 4-fold increase in FAR, which correlates with the symptoms of acute cholera. In addition to fluid accumulation, bloody mucus was observed in loops inoculated with the WT O139 strain, indicating haemorrhage. Similar observations were reported with WT O139 infections in a rabbit [55]. Our results indicate that both the LACV and unformulated VCUSM14P had no detectable diarrhoeagenic activity even at an inoculation dose of 10^4^–10^6^ CFU/mL in a rabbit ileal loop model without signs of haemorrhage and reactogenicity. A similar observation with VCUSM14 at doses of 10^6^ and 10^8^ CFU was reported by [44].

A RITARD assay was performed to further validate the results of the rabbit ileal loops assay. Rabbits vaccinated with the LACV formulation or unformulated VCUSM14P survived the challenge with WT O139 and showed no signs of diarrhoea or other symptoms of disease or death for up to 5 days of the observation period. However, unvaccinated rabbits developed cholera symptoms, and 100% mortality was observed in unvaccinated rabbits within 24 h post-challenge. Our RITARD results similar to those of *V. cholerae* ghost (VCG) vaccine candidate [56] and purified outer membrane vesicle [57] protection against virulent *V. cholerae* O1 (El Tor) and O139 strains. Similarly, our findings are also in agreement with protection by the live attenuated cholera vaccine VA1.4 against WT O139 in the RITARD model [30].

The excretion of vibrios in a vaccinated rabbit’s faecal material is a marker of intestinal colonization. Prolonged shedding of vibrios indicates successful colonization and subsequent multiplication of the vibrios in the small intestine [58]. Shedding of vibrios by the vaccinated rabbits after the first immunization indicates successful colonization of the vaccine strain, which correlates with our findings in the RITARD study and mouse colonization assay. No shedding of the vaccine strain was recorded after the booster dose. This result illustrates that the immune response induced by the first immunization was efficient enough to eliminate the bacteria used for the second immunization. This observation was supported by the lack of fluid accumulation in vaccinated rabbits, indicating the presence of sufficient anti-CT neutralizing antibodies to prevent fluid accumulation in the intestinal loops.

Several studies have demonstrated the cholera toxin-neutralising ability of anti-CT IgG and IgA antibodies when animals are challenged with live toxigenic *V. cholerae* [59, 60]. An increase in anti-CT IgG/IgA titres would protect the host from cholera by neutralizing CT [61]. The vibriocidal assay has been an indicator to measure the protective efficacy of a vaccine against cholera [30, 62]. A four-fold or greater increase in serum vibriocidal antibodies is known to confer protective efficacy of a cholera vaccine [47, 52, 63]. In the present study, the pre-immune sera of unvaccinated rabbits were recorded with a low vibriocidal antibody titres (baseline GMT of 10), which indicates that the rabbits were not previously exposed to *V. cholerae* or related organisms. However, after the booster dose in immunized rabbits, the vibriocidal antibodies were increased by 31-fold with the LACV and 14-fold with unformulated VCUSM14P. The results obtained in this study are comparable to those obtained by [56] in the evaluation of *V. cholerae* ghost (VCG) vaccine candidates, which induced a 20-fold increase in vibriocidal activity in the vaccinated rabbits. In all of the LACV-vaccinated rabbits, a significant increase in anti-CT IgG and IgA antibody titres from the baseline GMT of 10 to 17.12 and 72.67, respectively, was recorded at the first-week post-vaccination. At 3rd and 4th week, the rabbits elicited increased titres of anti-CT IgG and IgA antibodies. At 4th week, the LACV-vaccinated rabbits exhibited a 275-fold increase in anti-CT IgG and a 15-fold increase in anti-CT IgA antibodies compared to those of the rabbits vaccinated with unformulated VCUSM14P. Similar trends were reported [64] with killed thimerosal-free oral cholera vaccine against *V. cholerae* O1 Inaba. Overall, our results indicate that both the LACV and unformulated VCUSM14P are capable of inducing the humoral and vibriocidal immune responses against WT O139 and protected vaccinated rabbits, as evidenced by the RITARD results.

## Conclusion

A single-dose, live cholera vaccine provides long-term protection and is preferred over multiple doses of a killed vaccine for sustained immunological protection against WT *V. cholerae* infection. The cold chain-free LACV formulation recorded a higher colonization potential in the infant mouse colonization assay than the unformulated strain and was found to be non-reactogenic, as evidenced by the ligated rabbit ileal loop assay. The vaccine formulation is highly immunogenic, elicited higher antibody titres, high vibriocidal antibodies when compared to the unformulated strain and provided 100% protective efficacy in rabbits against WT O139 challenge. The developed prototype cold chain-free live oral cholera vaccine is first of its kind, and it would represent a great opportunity to increase its outreach for the global immunization programme at a competitive cost to reinforce the Water, Sanitation and Hygiene (WASH) services supported by WHO. The vaccine formulation will be further validated in an accredited Good Laboratory Practice (GLP) facility.

## Methods

### Bacterial strains and growth media

The live attenuated *V. cholerae* strain (VCUSM14P), wild-type *V. cholerae* O139 Bengal strain (WT 0139) and CIR314 were obtained from the Department of Medical Microbiology and Parasitology, School of Medical Science, Universiti Sains Malaysia (USM), Malaysia. These strains were maintained as glycerol stocks and revived on Luria-Bertani (LB) agar supplemented with polymyxin B (0.75 μg/mL). Thiosulphate-citrate-bile salts-sucrose (TCBS) agar was used as a selective medium for *V. cholerae*. All chemicals and reagents were purchased from Sigma unless otherwise stated.

### Experimental animals

BALB/c mice and New Zealand White adult rabbits bred in the animal house at USM were used in this study. Three- to five-day-old BALB/c suckling mice and healthy adult male rabbits weighing 2.0–2.5 kg were used for the experiments. The rabbits were housed in a stainless steel cage individually (30-in. width × 29-in. length × 18-in. height), provided with water and fed a normal rabbit pellet diet ad libitum. The rabbits were acclimatized to laboratory conditions for 1 week prior to the experimental protocol to minimize stress.

### Test cholera vaccine

A glass vial containing the LACV was drawn from the production lot, which was stored at room temperature (25 °C ± 2 °C and 60% ± 5% relative humidity) in the ICH-compliant stability chamber (Binder-KBF 115, Germany) for 140 days. The LACV is a liquid formulation consisting of 5 × 10^6^ CFU/mL of the VCUSM14P strain. Normal saline was used as a negative control. Serial dilutions of the LACV were made and plated onto LB agar and incubated for 16 h at 37 °C for enumeration of the bacterial population.

### Preclinical evaluation of colonization potential, reactogenicity, protective efficacy and immunogenicity of the LACV in animal models

**Fig. 4:**
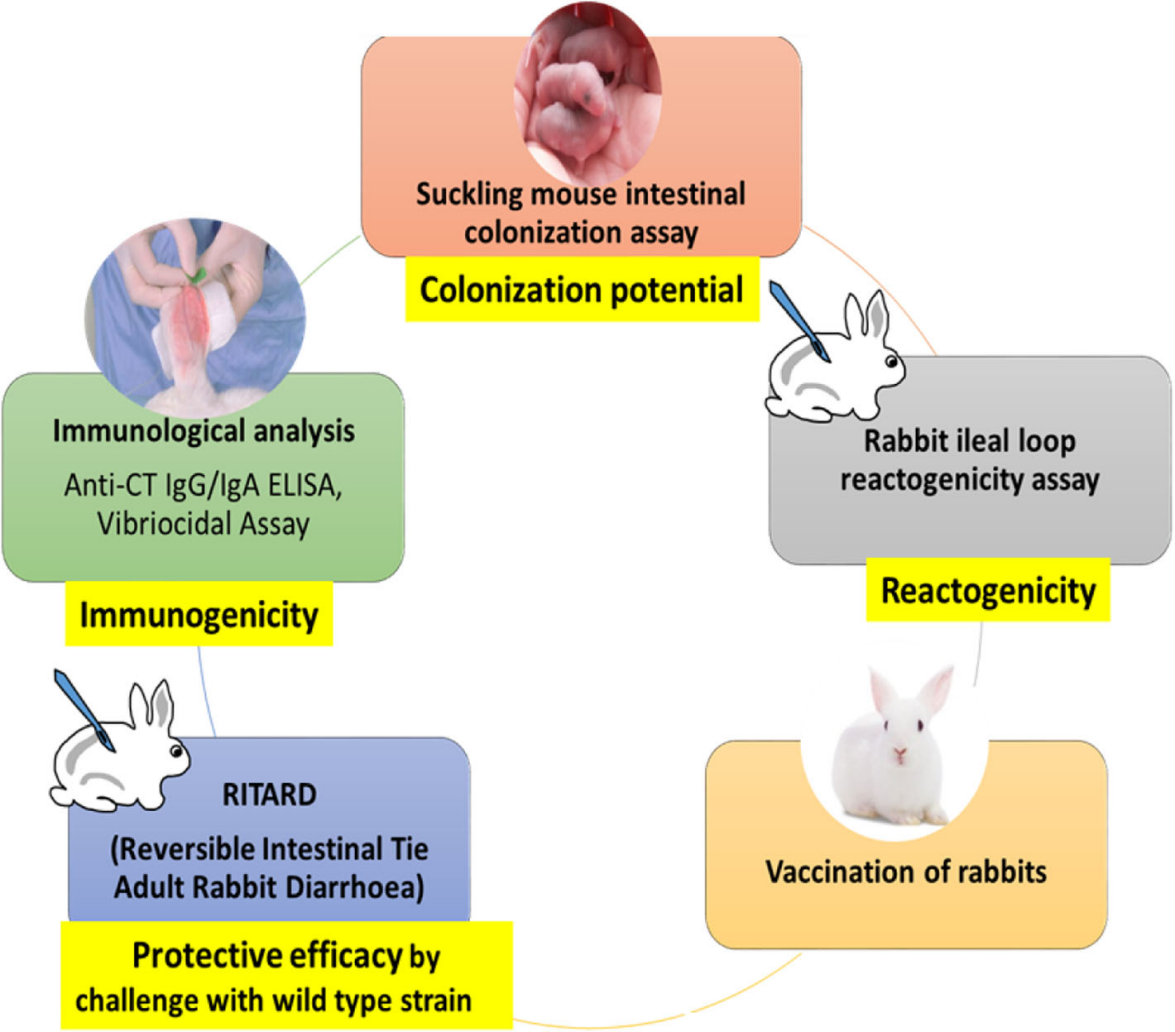
Flowchart showing different methods in the evaluation of colonization potential, reactogenicity, protective efficacy and immunogenicity of the LACV in animal models

The experiments were performed by five different methods for preclinical evaluation of the LACV as depicted in the flowchart (Fig. 4). The intestinal colonization potential of the LACV was determined in suckling mice. The reactogenicity and protective efficacy of the vaccine were evaluated in New Zealand rabbits. Immunological studies were carried out by performing anti-CT ELISA and vibriocidal assays.

### Suckling mouse intestinal colonization assay

*V. cholerae* colonization of the suckling mouse intestine is the most predominantly used host animal model to study *V. cholerae* pathogenesis in humans [61, 65–69]. A suckling mouse colonization assay was performed as described [44, 51, 70], with slight modifications to evaluate the colonization potential of the LACV. Forty mice were randomly divided into four experimental groups: G-I (normal saline), G-II (LACV), G-III (unformulated VCUSM14P) and G-IV (WT O139), each consisting of 10 mice per treatment group. The infant mice were fasted by separating them from their mother 1 h prior to inoculation. Each group of mice was administered with a suspension of the LACV, unformulated VCUSM14P, WT O139 and normal saline, respectively. Sterile Evans blue (1 μL) was added to the bacterial suspension as a colouring agent. Mice were administered intragastrically with 50 μL of the respective suspensions containing 2.5 × 10^5^ CFU. After 18 h post-inoculation, the mice were euthanized, and the intestines were excised. Viable vibrios in the gut were enumerated by plating dilutions of the homogenized whole intestine onto LB agar containing polymyxin B.

### Rabbit ileal loop reactogenicity assay

The ligated rabbit ileal loop assay was performed in unvaccinated rabbits as described [44, 51]. The two rabbits were fasted (water was provided ad libitum) for 24 h before the surgery. The rabbits were anaesthetized with ketamine (35 mg/kg) and acepromazine (1 mg/kg) administered intramuscularly. A midline incision was made along the linea alba approximately 5 cm in length. The ileum was exposed, and five-centimetre loops, each separated by 1 cm, were made by ligation using 2–0 silk suture. Each loop was injected with 1 mL containing 10^4^_,_ 10^5^ or 10^6^ CFU/mL of the LACV, unformulated VCUSM14P or WT O139, respectively. Normal saline (1 mL) was used as a control. After 18 h, the rabbits were euthanized with sodium pentobarbitone (100 mg/kg) intravenously, and the ligated loops were dissected. The length of the loop (cm) and the volume of accumulated fluid (mL) in each loop were measured. Reactogenicity was described as the fluid accumulation ratio (FAR). The FAR was obtained by dividing the volume of fluid (mL) accumulated per loop by the length (cm) of the respective loop.

### Immunization of rabbits with the LACV or unformulated VCUSM14P

Oral immunization of rabbits was carried out on day 0 and day 14 through the oro-gastric route as described by [44, 51]. Prior to vaccination, the 6 rabbits were orally administered 125 mg/kg of metronidazole. The rabbits fasted for 24 to 36 h before the first vaccination, but the water was provided ad libitum. The rabbits were anaesthetized with ketamine (35 mg/kg) and xylazine (4 mg/kg) by intramuscular administration. Cimetidine (50 mg/kg) was administered intravenously to each rabbit to reduce gastric acid secretion in the stomach. After 15 min, 15 mL of 5% sodium bicarbonate was administered twice intragastrically to neutralize stomach acid at 15-min intervals. Subsequently, rabbits were orally administered 10 mL of the LACV (5 × 10^6^ CFU/mL) or a suspension of unformulated VCUSM14P (5 × 10^6^ CFU/mL) in normal saline. After 30 min, 1 mL of morphine (10 mg) was injected intraperitoneally to slow peristaltic movements in the intestine. The rabbits were returned to their cages and provided with food and water. The second vaccination was carried out on the 14th day. The pre-immune and post-immune blood samples (5–7 mL) were drawn at an interval of 7 days, from day 0 up to 28 days post-immunization. Sera were obtained from coagulated whole blood by centrifugation at 1000 x g for 10 min and stored at − 20 °C until testing for the presence of antibodies.

### Protective efficacy in RITARD (reversible intestinal tie adult rabbit diarrhoea)

The RITARD assay was performed on the non-immunized and immunized rabbits as described before with minor modifications [44, 51, 71]. The rabbits were fasted for 24 h prior to the surgery and provided with water ad libitum. The rabbits were anaesthetized as described earlier. The caecum of the rabbit was ligated with 3–0 silk suture. A reversible knot was tied on the ileum 10 cm away from the ileocaecal junction with 2–0 catgut suture. Rabbits were challenged with 1 × 10^9^ CFU of toxigenic WT O139, which was injected into the jejunum 10 cm distal to the stomach. After 2 h, the temporary knot at the ileum was released, and the rabbits were monitored for any diarrhoea or mortality every 6 h for up to 5 days. On day 6, the rabbits were euthanized with sodium pentobarbitone (100 mg/kg) intravenously injected in a marginal ear vein. An autopsy was performed if the rabbit died within 5 days. On day 6, the surviving rabbits were sacrificed and checked for fluid accumulation and haemorrhage in the small intestine.

### Rectal swab

To detect the presence of viable VCUSM14P in the rabbit subjects, rectal swabs were collected every 24 h after the first and second vaccination, up to 5 days and during the RITARD challenge. The sterile cotton swab was moistened with alkaline peptone water and inserted 1–2 cm deep into the rectum of the rabbits. The rectal swab was spread on TCBS agar plates and incubated at 37 °C for 16 h to detect the presence of viable VCUSM14P cells.

### Immunological analysis

#### Anti-cholera toxin (CT) IgG and IgA ELISA

The immune response of rabbits immunized with the LACV or unformulated VCUSM14P was evaluated by measuring anti-CT IgG by ELISA as in [44, 51]. The ELISA plates (MaxiSorp, Nunc, Roskilde, Denmark) were coated with 0.5 μg/well of cholera toxin (Sigma, MO, USA) in 60 mM carbonate buffer (pH 9.6) and incubated at 4 °C for 16 h. The plates were then blocked with 5% skim milk and incubated at 37 °C for 1 h. The wells were washed 3 times with wash buffer (PBS - Tween 20) and 100 μL of each sera sample (1:10–1:1280 diluted in PBS) was added and incubated at 37 °C for 2 h. The plates were washed again with wash buffer and 100 μl of anti-rabbit IgG conjugated with HRP (Dilution 1:5000 in PBS) was added and incubated at 37 °C for 30 min. Subsequently, the wells were washed, and 2,20-azinobis (3-ethylbenzothiazoline-6-sulphonic acid (ABTS) was added as the substrate and incubated at 37 °C in the dark for 30 min. The absorbance reading was measured at 405 nm using 495 nm as the reference wavelength in a microtitre plate reader. The protocol was the same as described above except the primary anti-CT antibodies were captured with anti-rabbit-IgA-HRP diluted 1:3000 in PBS.

#### Vibriocidal assay

The immune response of rabbits immunized with either the LACV or unformulated VCUSM14P was evaluated by measuring vibriocidal antibodies as previously described with minor modifications [72, 73]. The serum samples were heated (at 56 °C for 30 min) to inactivate complement. A series of two-fold dilutions of serum samples in PBS was made (1:10 to 1:1280). The diluted serum samples (25 μL) were added to each well in a 96-well microtitre plate. The indicator strain for *V. cholerae* O139 (CIR 314) grown in LB broth overnight at 37 °C was diluted with PBS containing 20% guinea pig complement to a final concentration of 5 × 10^6^ cells/mL. This cell suspension (25 μL) was added to each well in the microtitre plate and incubated for 60 min. After 60 min, 150 μL of pre-warmed LB broth was added to each well and incubated for 4 h. The optical densities were measured at 600 nm with a microtitre plate reader. The vibriocidal antibody titre was defined as the highest serum dilution causing 100% killing of cells compared to the pre-immune sera.

#### Euthanization of rabbit

The rabbits were anaesthetized with ketamine (50 mg/kg) intramuscularly as described above. The rabbits were euthanized by injecting sodium pentobarbitone (100 mg/kg) intravenously at marginal ear vein. Euthanized rabbits were wrapped in a biohazard plastic bag and sent for incineration.

### Statistical analysis

The mean ± standard error of the mean (SEM) values was calculated for each group. All data were analysed using one-way ANOVA, followed by Tukey’s post hoc test. *P* < 0.05 was considered statistically significant.

## Data Availability

All data is available upon author request.

## Abbreviations

LMICs: Low-and middle-income countries
WHO: World Health Organization
ALA: Aminolevulinic acid
LACV: Live-attenuated oral cholera vaccine
ICH: International Council for Harmonisation
RH: Relative humidity
SD: Sprague Dawley
FAR: Fluid accumulation ratio
RITARD: Reversible intestinal tie adult rabbit diarrhoea
CFU: Colony forming unit
WT: Wild-type
ELISA: Enzyme-linked immunosorbent assay
VCG: *V. cholerae* ghost
CT: Cholera toxin
IgG: Immunoglobulin G
IgA: Immunoglobulin A
WaSH: Water, Sanitation and Hygiene
GLP: Good Laboratory Practice
LB: Luria-Bertani
USM: Universiti Sains Malaysia
TCBS: Thiosulphate-citrate-bile salts-sucrose
ABTS: 2,20-azinobis (3-ethylbenzothiazoline-6-sulphonic acid
SEM: Standard error of the mean
ANOVA: Analysis of variance
